# Effects of megavoltage computed tomographic scan methodology on setup verification and adaptive dose calculation in helical TomoTherapy

**DOI:** 10.1186/s13014-018-0989-y

**Published:** 2018-04-27

**Authors:** Jian Zhu, Tong Bai, Jiabing Gu, Ziwen Sun, Yumei Wei, Baosheng Li, Yong Yin

**Affiliations:** 1 Department of Radiation Oncology, Shandong Cancer Hospital and Institute, 440# Jiyan Road, Jinan, 250117 Shandong Province People’s Republic of China; 2 Medical Department, Affiliated Hospital of Shandong Academy of Medical Sciences, Jinan, 250031 People’s Republic of China

**Keywords:** TomoTherapy, Megavoltage computed tomographic, Image-guided radiotherapy, Adaptive radiotherapy

## Abstract

**Background:**

To evaluate the effect of pretreatment megavoltage computed tomographic (MVCT) scan methodology on setup verification and adaptive dose calculation in helical TomoTherapy.

**Methods:**

Both anthropomorphic heterogeneous chest and pelvic phantoms were planned with virtual targets by TomoTherapy Physicist Station and were scanned with TomoTherapy megavoltage image-guided radiotherapy (IGRT) system consisted of six groups of options: three different acquisition pitches (APs) of ‘fine’, ‘normal’ and ‘coarse’ were implemented by multiplying 2 different corresponding reconstruction intervals (RIs). In order to mimic patient setup variations, each phantom was shifted 5 mm away manually in three orthogonal directions respectively. The effect of MVCT scan options was analyzed in image quality (CT number and noise), adaptive dose calculation deviations and positional correction variations.

**Results:**

MVCT scanning time with pitch of ‘fine’ was approximately twice of ‘normal’ and 3 times more than ‘coarse’ setting, all which will not be affected by different RIs. MVCT with different APs delivered almost identical CT numbers and image noise inside 7 selected regions with various densities. DVH curves from adaptive dose calculation with serial MVCT images acquired by varied pitches overlapped together, where as there are no significant difference in all *p* values of intercept & slope of emulational spinal cord (*p* = 0.761 & 0.277), heart (*p* = 0.984 & 0.978), lungs (*p* = 0.992 & 0.980), soft tissue (*p* = 0.319 & 0.951) and bony structures (*p* = 0.960 & 0.929) between the most elaborated and the roughest serials of MVCT. Furthermore, gamma index analysis shown that, compared to the dose distribution calculated on MVCT of ‘fine’, only 0.2% or 1.1% of the points analyzed on MVCT of ‘normal’ or ‘coarse’ do not meet the defined gamma criterion. On chest phantom, all registration errors larger than 1 mm appeared at superior-inferior axis, which cannot be avoided with the smallest AP and RI. On pelvic phantom, craniocaudal errors are much smaller than chest, however, AP of ‘coarse’ presents larger registration errors which can be reduced from 2.90 mm to 0.22 mm by registration technique of ‘full image’.

**Conclusions:**

AP of ‘coarse’ with RI of 6 mm is recommended in adaptive radiotherapy (ART) planning to provide craniocaudal longer and faster MVCT scan, while registration technique of ‘full image’ should be used to avoid large residual error. Considering the trade-off between IGRT and ART, AP of ‘normal’ with RI of 2 mm was highly recommended in daily practice.

**Electronic supplementary material:**

The online version of this article (10.1186/s13014-018-0989-y) contains supplementary material, which is available to authorized users.

## Background

Daily repeatability of patient positioning is vital to the successful treatment outcome in radiotherapy. The megavoltage computed tomographic (MVCT) image quality from helical TomoTherapy (HT) has been proved sufficient for image-guided radiotherapy (IGRT) including tumor identification and setup verification [1]. Moreover, considering that MVCT has a reliable CT number to electron density calibration curve, MVCT has also been proved accurate to be used for calculating adaptive daily dose distribution which is an assurance of adaptive radiotherapy (ART) [2]. HT MVCT consisted of advantages in lower absorption dose and a larger imaging capacity (theoretically 40 cm reconstruction field of view (FOV) × 160 cm longitudinal scanning length) than C-arm based cone beam CT (CBCT) technology [3, 4]; thus making HT daily MVCT imaging safe in assessing patient treatment location and make on-line adaptive re-planning possible using the same imaging data sets.

In the process of MVCT image acquisition on TomoTherapy Operator Station (version 5.0, Accuray, Sunnyvale, CA), there are options for ‘acquisition pitch’ (AP) and ‘reconstruction interval’ (RI). AP is directly proportional to couch speed, scan duration and especially correlated to the patient absorption dose [5]. In the meanwhile, RI is much related to 3D reconstruction with varied slice thickness which will impact MVCT image qualities especially in the craniocaudal resolution.

There are still debates regarding to the effect of MVCT scan options on setup verification. Studies argued that there is no noticeable difference in image quality among different APs [6] or the issue could be ignored [7], while some studies concluded that pitch variation and longitudinal MVCT image resolution make statistically significant contribution to the MVCT- planning kilovoltage CT (KVCT) registration process [8, 9]. To the best of our knowledge, there is no study published on the effect of MVCT scan options on adaptive dose calculation. In order to investigate the quantitative effect of MVCT scan and reconstruction options on IGRT and ART, an experiment with two different phantoms was performed and the clinical effects were analyzed and presented in terms of image qualities, the accuracy for setup verification and dosimetry discrepancies.

## Methods

### CT simulation and planning on phantoms

**Fig. 1:**
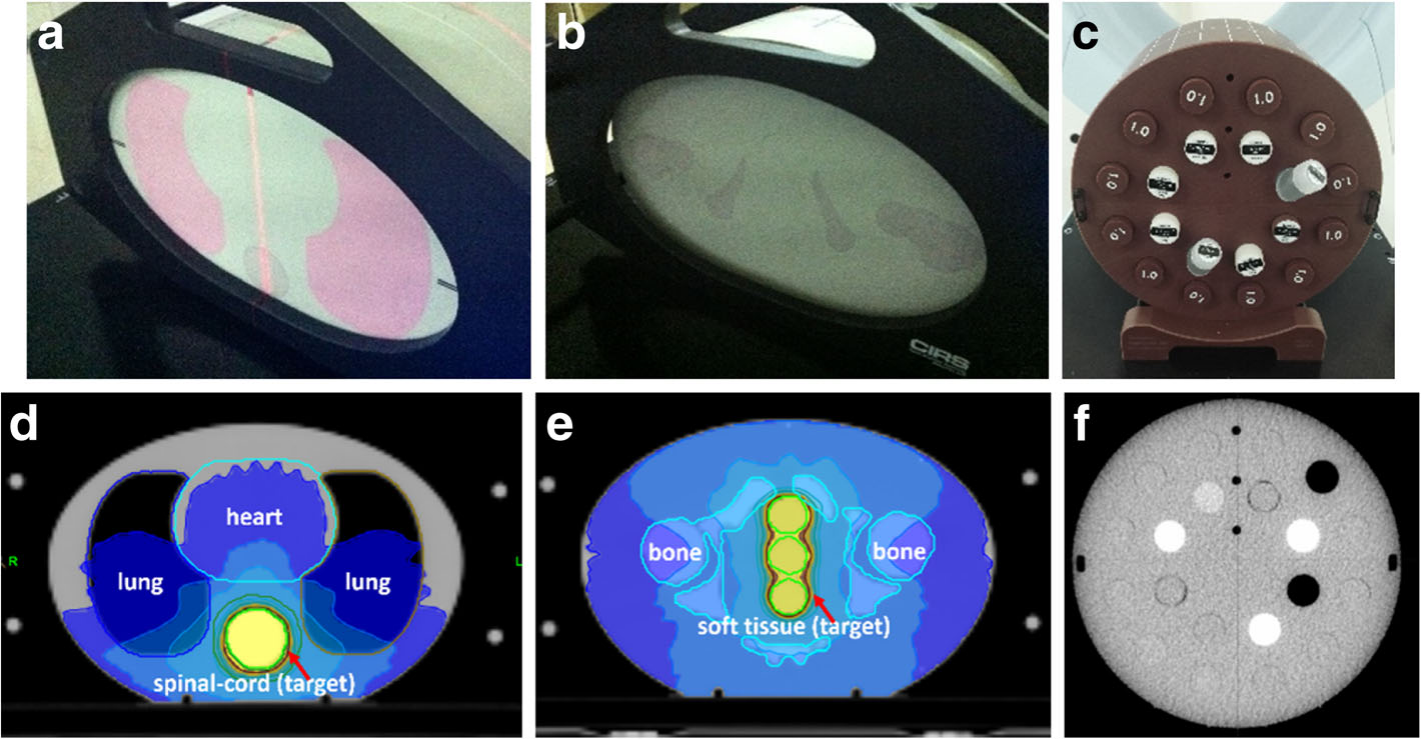
The transverse structures of the anthropomorphic heterogeneous chest (**a**), pelvic phantom (**b**) and the cylindrical ‘cheese’ phantom with 7 different densities of 20 sticks (**c**); the dose distributions on the simulated patient treatment plans where the yellow color wash stands for prescription dose on the simulated targets (**d**) (**e**); and the reconstructed transverse MVCT of the cylindrical phantom (**f**)

Anthropomorphic heterogeneous chest (Model 002LFC, CIRS, Norfolk, VA, USA) and pelvic phantoms (Model 002PRA) were used in this study (Fig. 1a,b). Three metal particles were placed on the surface of each phantom to serve as reference points for red laser alignment during planning and treatment setup. Spiral KVCT scans were performed for these phantoms on Philips Brilliance Big Bore CT (Philips, Cleveland, OH, USA) using a 3 × 3 mm stacked axial slice technique with a pitch of 0.938. The KVCT image resolution was 512 × 512 pixels. KVCT serials were transferred to Pinnacle (Philips Medical System, Eindhoven, Netherlands) treatment planning system (TPS) for contouring. Once the synthetic targets and normal tissues were all delineated on transverse sections, they were sent to TomoTherapy Physicist Station and the image resolution was downgraded to 256 × 256 pixels during this transfer process. To assess the dosimetry discrepancies among different MVCT serials, a simulated TomoTherapy treatment plan was mimicked on each phantom as shown in Fig. 1d, e. On KVCT with chest phantom (Fig. 1a), the spinal cord was contoured as a target to simulate a craniospinal irradiation (Fig. 1d); while with pelvic phantom (Fig. 1b), a region of soft tissue was contoured as a target to simulate a prostate irradiation (Fig. 1e). A prescription dose of 3Gy with one fraction was assigned to the simulated targets. The TomoTherapy planning parameters were as follow: dose calculate grid = fine, field width = 2.51 cm, modulation factor = 2.00 and pitch = 0.287.

### Phantoms setup and MVCT scan

First, the phantoms were set up in position without shift. For chest and pelvic phantoms, each was placed on TomoTherapy treatment couch respectively by aligning the metallic marker correlated to the red lasers. Then six series of MVCT images were obtained on helical TomoTherapy system with 6 groups of scan settings (Technical setting were listed in the left column of Additional file 1: Table S1). The groups consisted of 3 AP options of ‘fine’ (4 mm/rotation), ‘normal’ (8 mm/rotation) and ‘coarse’ (12 mm/rotation) multiplied by 2 with corresponding RI (fine: 1 and 2 mm; normal: 2 and 4 mm; coarse: 3 and 6 mm). The craniocaudal scan length and scan time were recorded for each MVCT scan. All MVCTs were transferred to TomoTherapy Physicist Station and Planned Adaptive module for adaptive dose calculation.

Secondly, setup with shift. After each chest and pelvic phantom was placed according to the red lasers and metal particle positions, the couch was manually shifted to X, Y, Z axial positive directions for 5.0 mm respectively by Positioning Control Panels which is located on the gantry of the machine with the precision of 0.1 mm in a cardinal IEC scale. Wherein positive direction denotes deviation in left (+X), superior (+Y) and anterior (+Z); while negative direction denotes deviation in right (−X), inferior (-Y) and posterior (−Z). Then transverse MVCT images were obtained again for each phantom with the above scan parameters respectively for the 6 serials. All the 6 serials of MVCT were also transferred to TomoTherapy Planned Adaptive module for offline image registration analysis.

In order to evaluate MVCT scan options on image quality, the cylindrical Virtual Water™ phantom (named ‘cheese phantom’) from TomoTherapy with 20 inserts of 7 various densities (Fig. 1c,f) was also scanned by all MVCT scan options for 6 serials. These series of MVCT images (Fig. 1f) were exported and the 7 different density plugs were contoured on each slice in Varian Eclipse treatment planning system (Varian Medical Systems, Inc., Walnut Creek, CA). The average CT numbers and image noises in the delineated regions of interest (ROIs) on the MVCT images were recorded.

The other MVCT acquisition factors during the TomoTherapy scan include jaw width projected to isocenter ~ 4 mm, gantry period 10 s/rotation, reconstructed FOV ~ 39 cm, irradiation energy of 3.5 MV and dose rate of 45 MU/min.

### Registration schemes

To investigate the accuracy of MVCT-KVCT registration, MVCT images were automatically registered by a modified mutual information algorithm called ‘extracted feature fusion’ with planning KVCT in the module of TomoTherapy Planned Adaptive for 6 serials, respectively. For any registration manipulation, four combinations of settings and their corresponding parameters, see Additional file 1, were available to choose from. The helical TomoTherapy system has 3 modes of rigid image registration: one based on bony anatomy, the other based on soft tissue, and the third based on bony anatomy as well as soft tissue, depending on the threshold applied to the Hounsfield numbers [10]. A previous study found that the bony anatomy-based auto registration is most useful [6]. While study from Suh argued that if MVCT provided better soft-tissue contrast, the soft-tissue image based registration would potentially improve setup accuracy [9]. Therefore, for the option of ‘selecting down sampling’ (three parameters), each was used respectively to test the involved pixels in the registration. For the option of ‘Uniform down sampling’, considering that down sampling will obviously affect the registration results [8, 11, 12] without significant time saving, the parameter of ‘Superfine’ was recommended to be applied. For the option of ‘Incomplete FOV’, it is not evaluated since both phantoms in this study will not extend outside of MVCT FOV. For the option of ‘rotational degrees of freedom’, the parameter of ‘translations only’ was selected since 96.6% of the rotational corrections were less than 4° and TomoTherapy treatment couch can only support translational movement in three dimensions [6].

### Evaluation methods and factors

Considering treatment efficiency, the scan time, craniocaudal scan length and reconstruction slice numbers of each MVCT series were recorded. Scatterplot and bar histogram were used to present the deviation in average CT number and image noise among 3 metrics of APs. Average CT number was calculated as the mean value of all pixels in each different density plug on the same transversal slice of MVCT image. Image noise was identified as the standard deviation of CT number divided by the mean value of all the pixels in the same density measurement area.

The simulated adaptive patient treatment plan of each phantom was recalculated on 6 MVCT data sets using TomoTherapy Planned Adaptive module. The corresponding dosimetric results were presented and compared with 6 groups of dose volume histogram (DVH) outcomes. Covariance statistical analysis was also used to evaluate the difference on intercept and slope of the DVH curves that were calculated on the most system elaborated (AP of ‘fine’ + RI of ‘1 mm’) and the coarsest (AP of ‘coarse’ + RI of ‘6 mm’) combinations MVCT data sets. Two curves are identified as different when covariance statistical analysis presents significant with *p* < 0.05. In order to encompass the whole phantom besides of certain ROIs, gamma index analysis method [13] was also used to compare ART dose distributions point by point on MVCTs from different APs by SNC Patient™ software (Sun Nuclear Corporation, Melbourne, FL). Parameters were set as follow: Threshold (TH %) 0.0%, which is the minimum dose percent value that must be met in either dose data from AP of ‘fine’ or dose data from AP of ‘normal’ and ‘coarse’ for the point to be included (0.0% means the whole dose distribution data in the entire image space) in the analysis; Diff(%) 1.0 and Dist 1.0 mm, that are dose difference tolerance and distance tolerance equal to or below which the gamma value of a point is considered ‘passing’ and beyond which a point is considered ‘failing’.

To mathematically evaluate the deviation between the calculated shift distance given by CT-MVCT rigid registration and the expected 5 mm shift distance, an equation of distance-to-agreement (DTA, unit millimeter) was defined as follow:

$$ \mathrm{DTA}=\sqrt{{D_x}^2+{D_y}^2+{D_z}^2} $$

where *D*_*x*_, *D*_*y*_, and *D*_*z*_ are the deviations between the registration result and the expected 5 mm shift distance on X (left-right), Y (superior-inferior) and Z (anterior-posterior) axis direction respectively. Theoretically, the registration result should ideally calculate as − 5.0 mm in each direction in order to meet the artificial 5.0 mm shift originally. Therefore, the smaller DTA value (close to 0) means the better registration result that indicates the lower effect of scan options on setup verification.

## Results

### MVCT scan analysis

Table 1 shows the phantom scanning time and reconstruction slice numbers in 6 groups of scanning settings in TomoTherapy MVCT IGRT system. For the same phantom, the scan time was basically inversely proportional to the AP, and the reconstruction slice number was inversely proportional to the RI. The scanning time of AP of ‘fine’ was approximately 2 times of ‘normal’ and 3 times of ‘coarse’, which is not affected by the RI options. The reconstruction procedure is independent of RI, which is fast enough to be taken as real-time.

**Table 1:** The scan time and reconstruction slice number at 6 groups of scan options on TomoTherapy MVCT image-guided radiotherapy system

Scan Options		Chest Phantom			Pelvic Phantom		
Acquisition Pitch	Reconstruction Interval	Scan Length (mm)	Scan Time (s)	Reconstruction Slices	Scan Length (mm)	Scan Time (s)	Reconstruction Slices
Fine (4 mm/r^a^)	1 mm	135	356	135	106	283	106
	2 mm	137	361	69	106	281	53
Normal (8 mm/r)	2 mm	136	191	69	106	151	53
	4 mm	134	186	34	107	151	27
Coarse (12 mm/r)	3 mm	139	136	47	108	108	36
	6 mm	137	131	23	114	111	19

^a^mm/r: the movement distance of treatment couch during one rotation of the beam gantry

### Effects on MVCT image quality

**Fig. 2:**
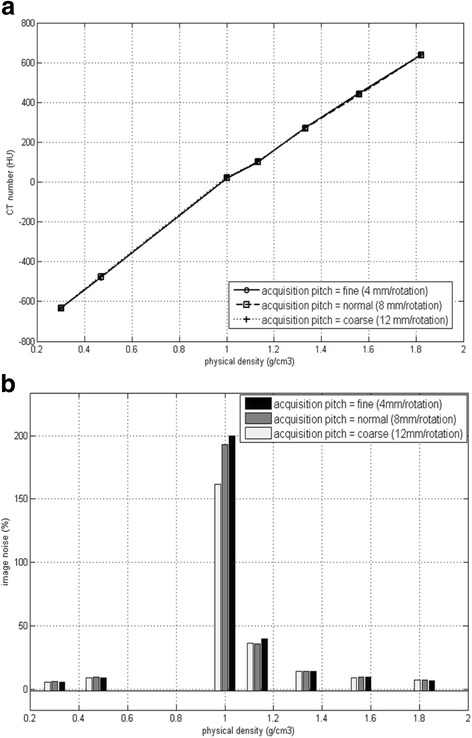
The relationship between CT number (**a**), image noise (**b**) and physical densities

Figure 2 shows the relationships between CT number, image noise and physical densities on 3 series of MVCT images. Since reconstruction interval is independent of transversal image performances, thus, only acquisition pith (AP) selections (fine, normal and coarse) were considered. Three sets of CT density curves were nearly overlapped in Fig. 2a indicating that MVCT acquisition with different APs have very similar CT numbers. In Fig. 2b, the discrepancy of MVCT image noise from three APs presents minimum significance in 7 selected density regions.

### Effects on dose calculation

**Fig. 3:**
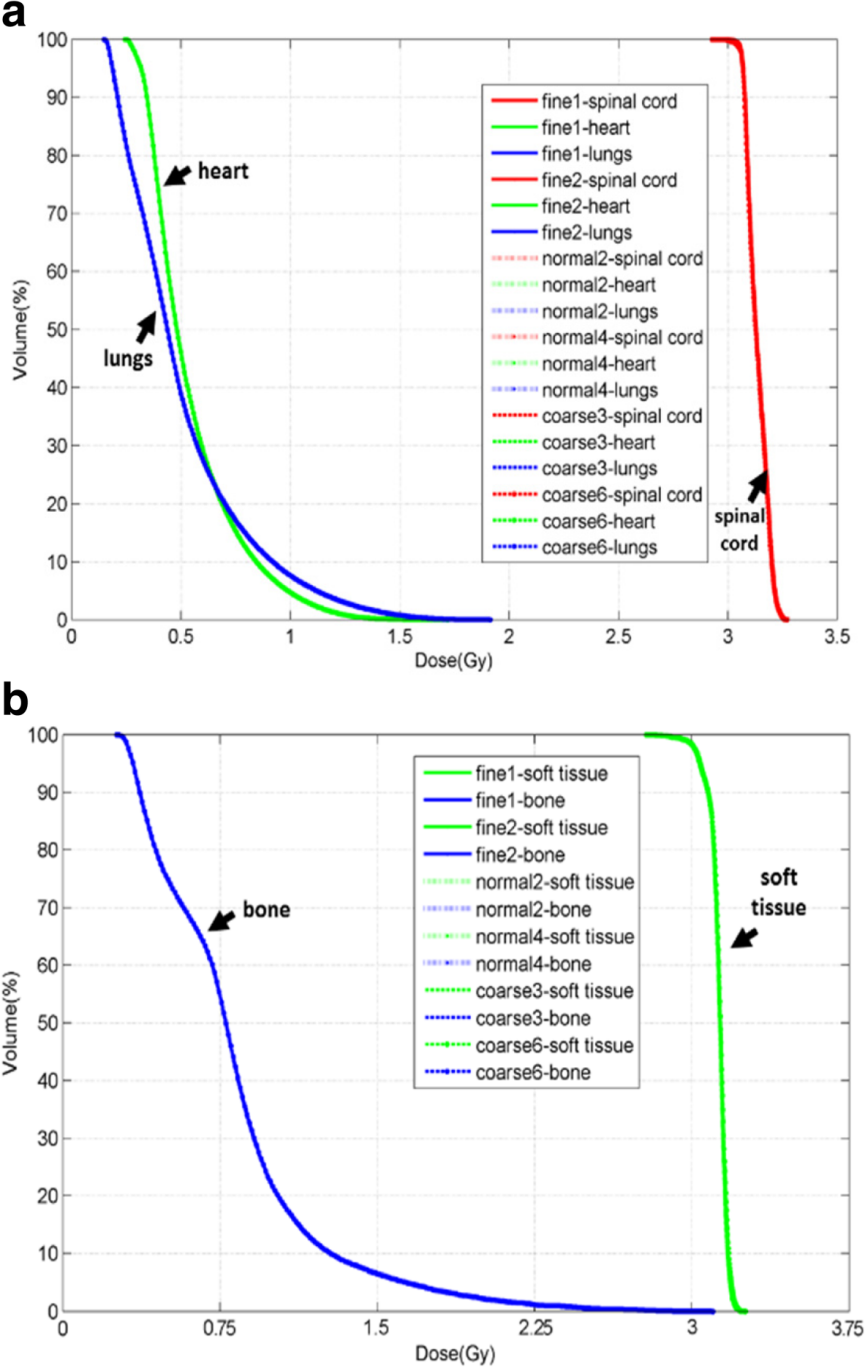
DVH for region of interests (ROIs) on chest (Fig. 3a) and pelvic (Fig. 3b) phantoms with the dose calculated on 6 series of MVCT images with arrows pointing to the anthropomorphic ROI in each phantom. The curve names in both legends of these two figures have the same structures. Take ‘fine 1 – spinal cord’ as example: ‘fine’ stands for the acquisition pith (fine, normal and coarse), ‘1’ stands for the reconstruction interval (1, 2, 3, 4 and 6 mm) and ‘spinal cord’ stands for one of the ROIs (spinal cord, heart, lungs on chest phantom, and soft tissue, bone on pelvic phantom) delineated on the MVCT image serials

DVHs with 5 allocated ROIs in the chest (Fig. 3a) and pelvic (Fig. 3b) phantoms were plotted in Fig. 3 with adaptive dose calculation in these 6 series of MVCT image sets. It was concluded that for all ROIs, each DVH curve calculated on different MVCT images overlapped together with no difference by objective observation. Analytical analysis indicated that the covariance statistical analysis result which aims to compare DVH curves were as follows: on both chest and pelvic phantoms, all *p* values of simulated spinal cord (*p* = 0.761 and 0.277), heart (*p* = 0.984 and 0.978), lungs (*p* = 0.992 and 0.980), soft tissue (*p* = 0.319 and 0.951) and bony structures (*p* = 0.960 and 0.929) were definitely present no major significance between the most elaborated and the coarsest MVCT imaging techniques.

**Fig. 4:**
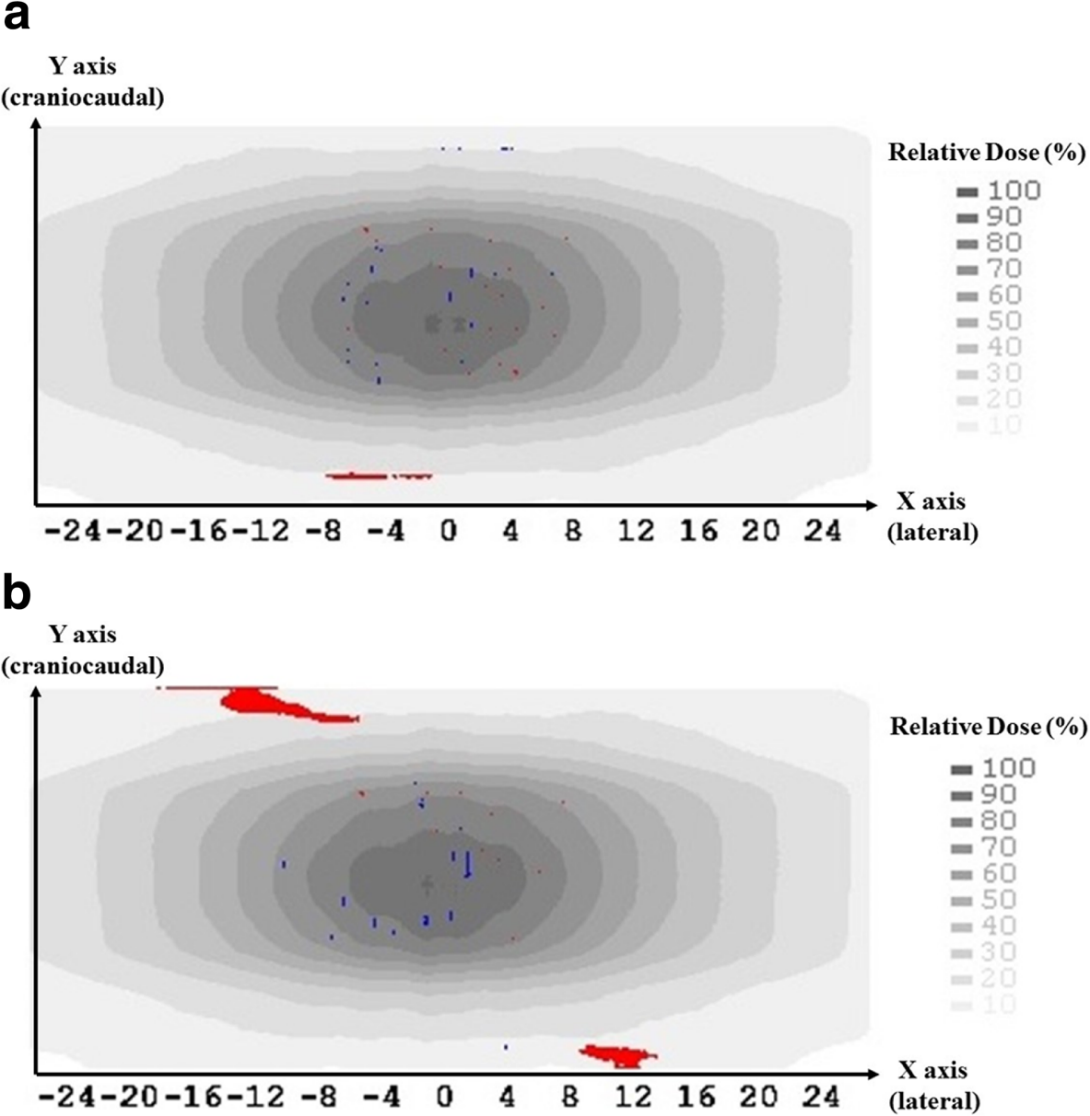
The gamma values that failed the comparison criteria (Threshold 0.0%, dose difference tolerance 1.0% and distance tolerance 1 mm) were colored for ‘fine vs. normal’ (**a**) and ‘fine vs. coarse’ (**b**) respectively. Red points indicate higher dose and blue points indicate lower dose from MVCT with AP of ‘fine’ compared to AP of ‘normal’ (**a**) and ‘coarse’ (**b**). X axis presents the lateral coordinate of the pelvic phantom and Y axis arrow points to the craniocaudal superior direction

Regarding to the gamma index analysis, a total 129,762 check points were included in the whole dose distribution space calculated on MVCT images of AP ‘fine’, ‘normal’ and ‘coarse’ with smaller RIs. After gamma values were checked point by point, passed ratio were 99.8% (129,491 points with gamma< 1) between MVCT AP of ‘fine’ and ‘normal’, and 98.9% (128,391 points with gamma< 1) between ‘fine’ and ‘coarse’. It indicated that only 0.2% or 1.1% of the points analyzed do not meet the defined gamma criterion. A 2-D plot of the gamma values that failed the comparison criteria (Threshold 0.0%, dose difference tolerance 1.0% and distance tolerance 1 mm) on the coronal center slice were colored for ‘fine vs. normal’ (Fig. 4a) and ‘fine vs. coarse’ (Fig. 4b) respectively. It also send the same message with above that, if MVCT ‘fine’ is considered as reference, fewer points from MVCT ‘normal’ failed the comparison than MVCT ‘coarse’. Fig. 4 also shows that MVCT image with larger AP may underestimate the low dose at the superior-inferior edge of the MVCT image space. However, most of the failed points appears on the low dose area (< 20% prescription dose), which is usually not considered and not presented during the planning process. Considering the standard y-jaw setting for MVCT imaging mode is fixed to 4 mm regardless of Aps option [14], the larger difference on dose calculation on the MVCT craniocaudal edge may attribute to faster couch moving during the imaging process. More experiments are expected to prove this assumption.

So far, both visual inspections as well as statistical factors indicated that the choices of AP and RI would not significantly impact the adaptive dose calculation, no matter which parameter was selected during the MVCT scan on TomoTherapy IGRT system.

### Effects on registration

**Fig. 5:**
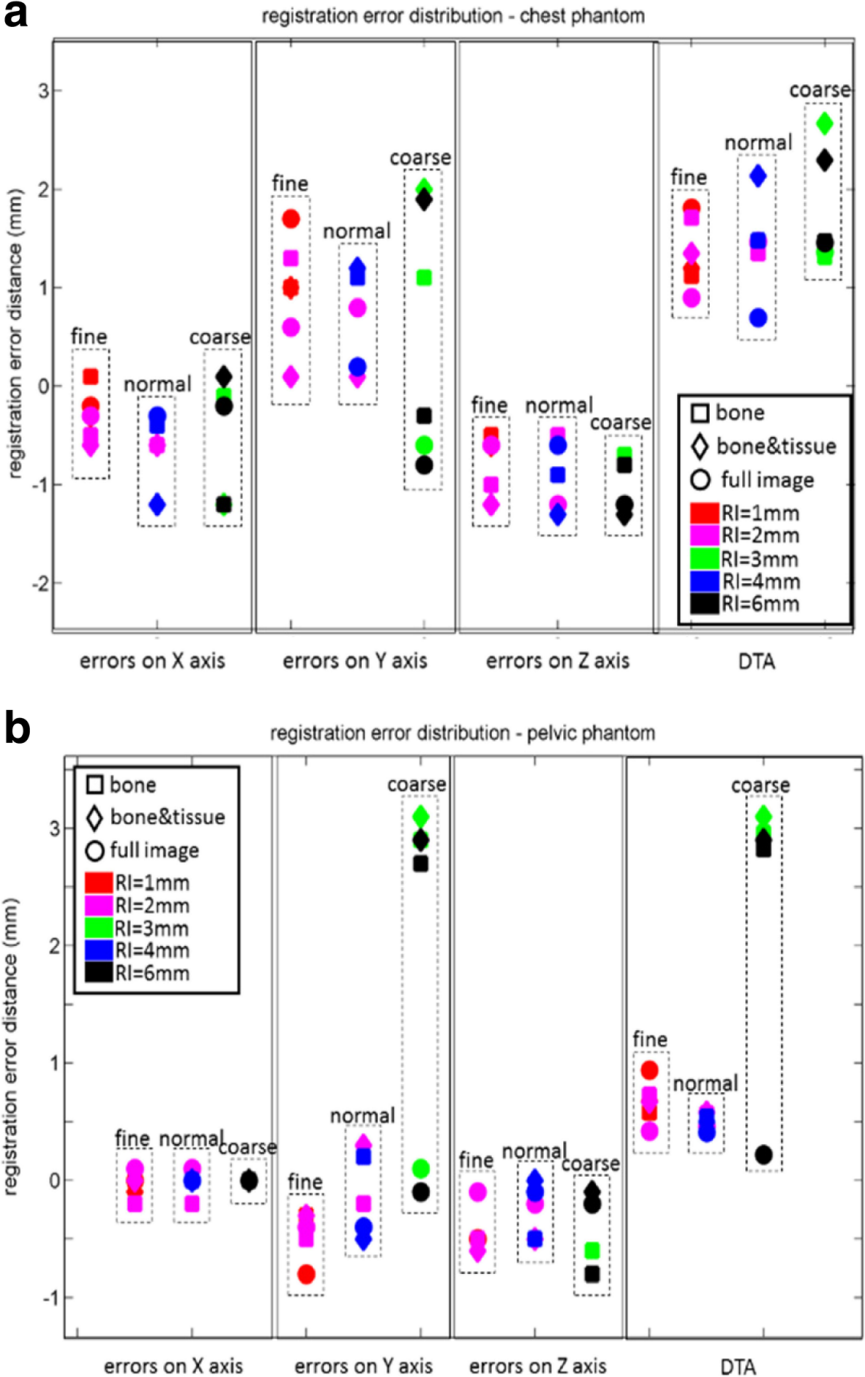
**a** The registration error (on axis X, Y, Z) and the DTA distribution, which comes from the discrepancy between the calculated shift distance given by CT-MVCT rigid registration and the expected 5 mm shift distance. **b** DTA describes the distance from the spacial position of registration error (Dx, Dy, Dz) to the origin of coordinates (0, 0, 0). ‘fine, normal and coarse’ denote the options of MVCT acquisition pitches. Different colors stand for different reconstruction intervals. Square, diamond and circle markers stand for different registration technologies

Figure 5 shows the registration errors (on axis of X, Y, Z respectively) and the DTAs distribution, which was generated from the discrepancy between the calculated shift distance given by CT-MVCT rigid registration and the pre-defined 5.0 mm translational manually shift.

Figure 5a present the results with chest phantom showing that: first, all registration errors > 1 mm appear on axis of Y (superior-inferior); secondly, even using the smallest AP (fine) and RI (1 mm) could not avoid relatively large craniocaudal errors (> 1 mm); thirdly, in term of DTA estimation, the larger AP would bring larger registration errors. Since each transversal MVCT slice of the chest phantom was almost identical (shown in Fig. 1d), it was assumed that the low and limited craniocaudal spatial resolution effect caused the major errors on axis of Y, which were larger than axis of X, and Z, inherently.

Figure 5b shows the results from pelvic phantom: first, as assumed above, craniocaudal errors were much smaller than that from chest phantom because the craniocaudal resolution of pelvic phantom is higher with its simulated bony structures; second, larger errors and DTA (about 3 mm) were obvious with AP of ‘coarse’; however, the ‘full image’ registration technique will reduce the large errors caused by AP of ‘coarse’ from 2.90 mm to 0.22 mm.

Comparing the results from both chest and pelvic phantoms, the AP of ‘normal’ option present good performance with less scan time and comparable registration accuracy to pitch of ‘fine’. In all cases, there is no significant difference on image registration accuracy for RI option, no matter it is along with AP of small (fine) or large (coarse).

## Discussion

IGRT is an efficient tool to improve the accuracy of targeting and is necessary to ART [15]. In head and neck and prostate cancer patients, setup error > 5 mm can occur in 10% [16] and 20% [4] fractions respectively when volume imaging was performed every second day. Li found that the interfractional variations in patient setup and in shapes, sizes, and positions of both targets and normal structures are site specific and may be used to determine the site-specific margins, which emphasized the importance of ART [17]. Duma [18] recommends daily MVCT scan for patients treated with doses close to the tolerance dose of the critical ROI (such as spinal cord). Furthermore, random setup errors can only be eliminated through the use of daily image guidance, whilst the dose from MVCT scan is critical to evaluate the treatment outcome. Quantitatively, Shah [4] proved that the typical HT MVCT imaging dose is approximately 1.5 cGy per image and the uniform MVCT dose delivered using HT is greatest when the anatomic thickness is the smallest and the pitch is set to the lowest value. Since reduplicative MVCT scans on human body is unethical, an anthropomorphic phantom based experiment was created for this study, which aims to give a reasonable MVCT scan protocol by investigating the effect of scanning setting and technique during setup verification and adaptive dose calculation in TomoTherapy system.

Since option of AP is directly proportional to scan time and creating additional irradiation dose to patients [5], it is recommended to use pitch as large as possible if there is no impact on IGRT imaging quality and ART accuracy. In term of adaptive dose calculation, our study shown that MVCT from any AP and RI combinations has no significant effect on these MVCT calculated dose distributions. Langen [2] has indicated that the MVCT pitch ratio has little effect on dose recalculation. The robustness of the recalculations is impressed, given the coarse sagittal reconstructions that were used [2]. With the conclusion, then the use of AP of ‘coarse’ may be tempting in an effort to reduce the imaging dose, which a patient has to receive due to the MVCT acquisition. Yue [19] also argued that different APs have no significant difference on CT number (*p* = 1.000) and image noise (*p* = 0.667) among MVCT imaging technical settings. But there is no study, to the best of our knowledge, has ever reported the effect of MVCT RIs. According to our study, we have concluded and recommended the fastest and harmless scan option in case of adaptive planning: AP of ‘coarse’ with RI of 6 mm. In term of ART, the image stability is another critical issue. As Langen [20] resulted that, during a 9-month period, an average variation of 20 Hounsfield units was seen (by a CT-to-electron density phantom) for the fan-beam MVCT imaging system. However, Langen [2] also argued that, this variation does not affect the dose calculation result significantly. An agreement of 0.5% was found between the planned and recalculated dose received by 95% of the target volume. Therefore, it was concluded that the fan-beam MVCT dose calculation accuracy is similar to that of the initial diagnostic KVCT dose calculation. And in another perspective, to approve the stability, MVCT image quality is one of the most important content in the Tomotherapy monthly QA protocol according to report TG-148, where image noise, uniformity, spatial resolution, contrast, and the MVCT dose are monitored. Furthermore, ‘CT number calibration’ is a mandatory weekly QA procedure on TomoTherapy operation station, which will compare the average measured HU to expected HU for both homogeneity water equivalent cheese phantom and air. If the absolute difference is smaller than 70 HU, TomoTherapy system will automatically correct the CT numbers in order to ensure consistency of the CT number values. So far, based on the arguments above, we can be confident to the MVCT image’s stability for accurate dose calculation in adaptive radiotherapy.

However, in term of IGRT, our study shows that large AP (coarse) at KVCT-MVCT registration would lead to large error, which was coincident to a previous study which found that 4 and 2 mm inter-slice spacings generally have lower residual error than 6 mm spacing [21] Therefore, to tradeoff between IGRT and ART requirement, AP of ‘normal’ with RI of 2 mm is recommended in daily IGRT and ART clinical application based on our research results. However, in case of stereotactic body radiation therapy (SBRT) with early stage lung tumor (< 4 cm) [22], since AP of ‘normal’ and ‘coarse’ have larger step size per rotation (8 mm/rotation and 16 mm/rotation, respectively) which may lose critical target anatomy information, tumor density [23] and texture features [24] which may be used to predict therapeutical effect, AP of ‘fine’ (4 mm/rotation) with RI 1 mm setting is recommended.

Smaller RI may improve the longitudinal display resolution, with neither impact on scan time, patient dose nor impact on ART dose recalculation as well as IGRT registration accuracy. Furthermore, larger reconstruction resolution may miss smaller tumor or lymph nodes in adaptive delineation and replanning process. Levegrun [8] also reminded that even with the finest pitch, the MVCT images of a thin object are blurred longitudinally to limit the visibility of small anatomic structures. Therefore, no matter which AP is selected, the smaller RI is recommended to provide relative higher resolution in daily IGRT and ART applications. However, since the maximum number of slices allowed for an MVCT scan is 300, thus the ‘coarse’ setting and larger RI is a compromise if the scan is necessary to cover as long as possible. In this case, ‘full image’ registration technique should be used to remedy the disadvantage of large AP that might generate large registration errors. A reasonable explanation is that registration with ‘full image’ technique enrolled more voxels from MVCT, which provided closer axial resolution to planning CT. Our recent studies also proved that, if two series of images have a closer axial resolution, the registration will be matched better and will deliver higher registration accuracy [12, 25].

Compared to lateral and ventrodorsal directions, our study shows that registration error in craniocaudal direction is significantly larger, which is consistent with previous published data and our previous study from both KVCT-MVCT and KVCT-CBCT registration techniques [8, 26–29]. This phenomenon may be caused by the trade-off against imaging speed and increase with the pitch of the MVCT scan [30]. Regarding to the craniocaudal registration errors, we found that it decreased in the pelvic phantom than in the chest phantom and we assumed it may come from the lower craniocaudal resolution in the chest but rather higher one in pelvis because of the complicated pelvic bony structures. Considering that the bony structures of the chest are similar in between transversal slices, the CT-MVCT registration protocol of craniospinal irradiation should probably be different from the other anatomic site such as head and neck and pelvic etc. Therefore, we suggest the craniocaudal scanning length should encompass at least one entire vertebra (including thoracic spine or lumbar spine) in craniospinal irradiation in order to correct the body roll direction and this claim should be substantiated by further research. Furthermore, the automatic registration algorithm could potentially eliminate inter-observer’s deviation altogether but also can impair local matches highly weighted by individual observers and may introduce a procedure-dependent new error [8]. Therefore, manually perform a double check and adjustment to the automatic registration result by radiation oncologist should be part of the strict CT-MVCT registration protocol in craniospinal irradiation, lest low craniocaudal resolution of MVCT may bring large registration error just as one vertebra is incorrectly matched to the adjacent one [30, 31].

MVCT scan takes approximate 10 s for one gantry rotation regardless of AP options, which will include 2~ 5 respiratory cycles for chest imaging. With the highest pitch, AP of ‘coarse’ will cover craniocaudal12mm per rotation. Considering that each fraction of scan should encompass the motion envelope of the target for accurate IGRT and ART, inspiratory motion and resultant imaging artifacts cannot be avoided by breath holding in TomoTherapy MVCT serials. A phantom based experimental study has proved that motion artifacts in TomoTherapy MVCT or planning CT studies changed the accuracy of the automatic registration process by less than 2.0% [21]. Based on this result, effects of AP and RI on IGRT and ART accuracy were investigated by static phantoms in this study, and we propose that it is possible to apply present findings to the moving targets or organs. However, this conclusion relies on two assumptions: first, moving target is much smaller than the total volume of the thorax imaged; second, respiratory movement of the organs like ribcage, diaphragm does not affect the registration process. If they cannot be met, gross motion may bring unexpected impact to IGRT and ART accuracy, which should be investigated by further research.

One of the probable resolution of gross respiratory motion and target shape change is deformable registration. As mentioned above, since manually performed double check is critical to accurate IGRT, a clinician may modify the automatic registration result based on their judgement on the alignment of the target. This may bring a new tradeoff between alignment of target and surrounded organs. Deformable registration can enroll more voxels and multi scales of mutual information during the match, which may meet the requirements from both target and organs, and bring more accurate registration in real situations. If so, we assume that AP of ‘fine’ with smaller RI may bring better deformable registration result and fewer residual errors because of its more sufficient imaging information. However, this assumption should also be substantiated by further research.

Beside of the translational corrections, TomoTherapy IGRT system can use the gantry angle to correct for the roll (along with the craniocaudal axis) adjustment automatically. TomoTherapy software allows a roll correction of any angle bigger than 0.1° (0.1° is the limiting value of gantry’s mechanical precision) prior to treatment. Rotational adjustment in one direction results in gantry rotation in the opposite direction to achieve the correct angular relationship between the radiation source and patient. However, roll correction was not enrolled in this study. We considered that if the image volume is rotated clockwise or counterclockwise, lateral and vertical adjustments become linked, and future lateral or vertical adjustments will proportionally affect each other. Since this study predominantly aimed to reveal the residual errors on 3 translational directions, rotational disagreement was eliminated to avoid its translational impacts in the experiment. However, if deformable registration method can be used in TomoTherapy IGRT module, rotational disagreement value and the corresponding correction may bring higher accuracy, which should also be substantiated by further research.

## Conclusions

From our study, AP of ‘coarse’ with RI of 6 mm is recommended in ART planning to provide craniocaudal longer and faster MVCT scan, while registration technique of ‘full image’ should be used to avoid large residual error. Considering the trade-off between IGRT and ART, AP of ‘normal’ with RI of 2 mm was highly recommended in daily practice.

## Additional files

Additional file 1: **Table S1.** Automatic registration options and parameters in TomoTherapy Planned Adaptive module. (DOCX 22 kb)

## Abbreviations

AP: Acquisition pitch
ART: Adaptive radiotherapy
CBCT: Cone beam CT
DTA: Distance-to-agreement
DVH: Dose volume histogram
FOV: Field of view
HT: Helical TomoTherapy
IGRT: Image-guided radiotherapy
KVCT: Kilovoltage computed tomographic
MVCT: Megavoltage computed tomographic
RI: Reconstruction interval
ROI: Regions of interest
TPS: Treatment planning system
