# Supplementary material for: Effects of megavoltage computed tomographic scan methodology on setup verification and adaptive dose calculation in helical TomoTherapy
Source: Radiat Oncol. 2018 Apr 27;13:80. doi: 10.1186/s13014-018-0989-y (PMC5921977; doi:10.1186/s13014-018-0989-y)
Supplement: Supplementary file 1 — Table S1. Automatic registration options and parameters in TomoTherapy Planned Adaptive module. (DOCX 22 kb) [file 13014_2018_989_MOESM1_ESM.docx]

**Additional file 1**

**Table S1** Automatic registration options and parameters in TomoTherapy Planned Adaptive module

| Automatic Registration Options | Parameters | Explanation |
| --- | --- | --- |
| Selective down sampling | Full Image | Mutual Information with no threshold |
|  | Bone and Tissue | Threshold to identify bone and tissue pixels (> 0.3 g/cm^3^), then register |
|  | Bone | Threshold to identify bone pixels (> 1.1 g/cm^3^), then register |
| Uniform down sampling | Superfine | No down sampling |
|  | Standard | Down sample 4x in R/L^*^, A/P^†^ directions |
|  | Fine | Down sample 2x in R/L, A/P directions |
| Incomplete FOV | Yes or No | Check this box to reduce the influence of artifacts on the registration if patient extends outside MVCT image FOV |
| Rotational degrees of freedom | Translations Only,  Translations+Roll,  Translations+Yaw, Translations+Yaw+Roll, Translations+Pitch+Yaw+Roll | Which degrees of freedom will be used? |

* R/L: Right/Left † A/P: Anterior/Posterior
